# Reliability of the active knee joint position sense test and influence of limb dominance and sex

**DOI:** 10.1038/s41598-022-26932-2

**Published:** 2023-01-04

**Authors:** Aglaja Busch, Christian Bangerter, Frank Mayer, Heiner Baur

**Affiliations:** 1 Sports Medicine & Sports Orthopeadics, University Outpatient Clinic, University of Potsdam, Am Neuen Palais 10, Building 12, 14469 Potsdam, Germany; 2 Division of Physiotherapy, Department of Health Professions, Bern University of Applied Sciences, Murtenstrasse 10, 3008 Bern, Switzerland

**Keywords:** Neuroscience, Physiology

## Abstract

The output of a sensorimotor performance can be measured with the joint position sense (JPS) test. However, investigations of leg dominance, sex and quality measures on this test are limited. Therefore, these potential influencing factors as well as reliability and consistency measures were evaluated for angular reproduction performance and neuromuscular activity during the active knee JPS test in healthy participants. Twenty healthy participants (10 males; 10 females; age 29 ± 8 years; height 165 ± 39 cm; body mass 69 ± 13 kg) performed a seated knee JPS test with a target angle of 50°. Measurements were conducted in two sessions separated by two weeks and consisted of two blocks of continuous angular reproduction (three minutes each block). The difference between reproduced and target angle was identified as angular error measured by an electrogoniometer. During reproduction, the neuromuscular activity of the quadriceps muscle was assessed by surface electromyography. Neuromuscular activity was normalized to submaximal voluntary contraction (subMVC) and displayed per muscle and movement phase. Differences between leg dominance and sex were calculated using Friedman-test (*α* = 0.05). Reliability measures including intraclass correlation coefficient (ICC), Bland–Altman analysis (bias ± limits of agreement (LoA)) and minimal detectable change (MDC) were analysed. No significant differences between leg dominance and sex were found in angular error and neuromuscular activity. Angular error demonstrated inter-session ICC scores of 0.424 with a bias of 2.4° (± 2.4° LoA) as well as MDC of 6.8° and moderate intra-session ICC (0.723) with a bias of 1.4° (± 1.65° LoA) as well as MDC of 4.7°. Neuromuscular activity for all muscles and movement phases illustrated inter-session ICC ranging from 0.432 to 0.809 with biases between − 2.5 and 13.6% subMVC and MDC from 13.4 to 63.9% subMVC. Intra-session ICC ranged from 0.705 to 0.987 with biases of − 7.7 to 2.4% subMVC and MDC of 2.7 to 46.5% subMVC. Leg dominance and sex seem not to influence angular reproduction performance and neuromuscular activity. Poor to excellent relative reliability paired with an acceptable consistency confirm findings of previous studies. Comparisons to pathological populations should be conducted with caution.

## Introduction

Sensory information plays an important role in human movement control and performance. The integration and processing of sensory information is a prerequisite for goal-directed movement, and for adjustment to external influencing factors (e.g., perturbations)^1^. A fundamental component of sensory information is proprioception^2^. Proprioception is defined as perception of motion, body segment position and force or tension^3^. This information among other sensory and motor capacities, as well as central integration and processing, comprises the sensorimotor system. The output of this system is a goal-directed and adjustable movement which can be quantified e.g., biomechanically. One possible method to assess the output is the active joint position sense (JPS) test^4^.

The JPS test examines the ability to reproduce a prior presented angle by a body segment^5^. The assessment can be conducted under active or passive conditions. The body segment is moved to a target angle and returned to the starting position either actively by the participants themselves or passively by an examiner. Afterwards, the participants are asked to reproduce the targeted angle either actively or passively^6^. Differences between the targeted and replicated angles display the accuracy of reproducibility and are defined as angular error ^7^. Furthermore, the JPS test can include either ipsilateral or contralateral movements. In the ipsilateral task, participants are asked to reproduce the target angle using the same body segment as initially shown. Conversely, in the contralateral test, the reproduction of the target angle is performed with the contralateral body segment^7^. Varying detection methods such as image capture, electrogoniometer, or dynamometer are used to record angular changes. There is a debate in the literature regarding their benefits and disadvantages, mostly reflecting aspects of the underlying basic measurement method^6^.

Besides different methodological approaches, confounding variables are also discussed in the literature. Physical activity or age seem to influence knee joint position sense^8,9^. Effects of leg dominance or sex have been published with various results in the literature^10,11^. Additionally, limited studies investigated the reliability and consistency of an active-active knee JPS in healthy participants ^6^. This might be the reason for diverse results on the influence of leg dominance and sex^12^. Therefore, to contribute and expand current knowledge, the focus of the present manuscript was on the influence of leg dominance as well as sex and the corresponding quality measures of the assessments.

Moreover, studies have revealed sensorimotor alterations after joint injury, e.g., anterior cruciate ligament (ACL) rupture and reconstruction^5,13^. It is assumed that these alterations are related to impaired muscle functionality and diminished activity level or balance abilities after ACL injury^14,15^. Thus, especially in the early phase of rehabilitation, sensorimotor tests can be used as outcome measures of improvement after joint injury or diseases^5,12^. To the authors knowledge no study has previously focused on muscle activation patterns during an active knee JPS test. Considering the aforementioned possible impaired muscle functionality after joint injury, it would be valuable to investigate neuromuscular activity during the execution of sensorimotor tests. Accordingly, evaluation of the parameters in healthy participants might give a reference for a later comparison to a pathological group.

A valid and reliable methodological approach is critical when assessing the effectiveness of interventions or to monitor progress^16^. Therefore, the objective of this study was to examine the influence of leg dominance and sex on outcomes of an active knee JPS test, and to assess the reliability and consistency of the angular reproduction performance as well as the neuromuscular activity during an active knee JPS test.

## Methods

### Participants

Twenty healthy adults voluntarily participated in this study. Participants were included if they were between 18 and 50 years of age, physically active (at least twice for 45 min per week^17^) and had no former knee pathology. Exclusion criteria were: cardiac, neurologic or peripheral vascular diseases, acute infection, alcohol abuse, current pain medication, other injury of the lower extremity or trunk, back pain, thrombosis, pregnancy, dementia, or other musculoskeletal disorders limiting successful execution of the test protocol.

### Procedure

After clarification of study inclusion, anthropometrical data, physical activity data (in minutes per week), Tegner activity Scale (TAS)^18^ and leg dominance of the participants were obtained. The dominant leg was defined as the self-reported leg the person would kick a ball with^19^. Subsequently, the participants were prepared for the neuromuscular activity measurement using electromyography (EMG; see EMG recordings and analyses). Followed by a standardized warm-up of 6 min level walking on a treadmill (Kettler Marathon TX 1, Kettler, Ense-Parsit, Germany) at a speed of 3 km*h^−1^. During the end of the warm-up, the neuromuscular activity was recorded to serve for later normalization of the EMG signals^20^.

For the JPS test, an electrogoniometer (Potentiometer RP20, Megatron Elektronik GmbH & Co. KG, Munich, Germany) was attached to the participants’ leg to quantify angular reproduction. The centre of rotation was placed at the knee gap, in the middle between lateral femoral and tibial epicondyle. The goniometer arms were attached in a superior/inferior orientation, aligned proximally with the greater trochanter and distally with the lateral malleolus. Participants sat with a hip angle of 100° flexion, with approx. a 5 cm gap between the knee pit and chair surface, and an initial knee angle of 90° flexion (0° = full extension), in an open kinetic chain. First, the participants actively extended their leg to the target angle of 50° knee flexion (movement range of 40°) according to the instruction of the examiner and visual feedback of the knee angle on a screen. Afterwards, the participants were asked to practice reproducing the angle in 5 familiarization trials. Visual feedback on a screen was also provided during this practice phase. However, the participants’ vision towards the legs was blocked with a hanging curtain during execution of the familiarization trials and the following testing. Two blocks of 3-min long continuous angular reproduction at a self-selected pace were executed (numbers of repetitions N = range of 19–92). The instruction was to reproduce the target angle as accurately as possible, hold it for three seconds and then return to the starting position (90° flexion). During the execution of the task, the neuromuscular activity and the angular changes were recorded. A 3-min break was taken between the blocks and measurements were performed with both legs, whereby the selection of the starting leg was randomized for each session. Participants were measured twice with a separation of two weeks.

### Electrogoniometer recording and processing

Electrogoniometer data were recorded at 4000 Hz and an analogue/digital conversion was conducted (NI PCI 6255, National Instruments®, Austin, USA; 1.25 MS/s, 16 Bit) before the signals were registered via LabVIEW®-based software (Pfitec®, Endingen Germany). Processing of the electrogoniometer data were performed in Imago Process Master (Pfitec®, Endingen, Germany). The reproduced angle of each trial was determined as the midpoint of the 3-s holding phase as described in the procedure description. The obtained angles were exported to a Microsoft® Excel spreadsheet (Windows 10, Microsoft Corporation, Redmond WA, USA) and further processed in R (R Core Team, version 4.1.0, 2021)^21^. The difference between the targeted and reproduced angle was calculated as angular error. Hereby, the constant angular error (CE), defined as error with directional bias (positive or negative arithmetical difference), and absolute angular error (AE), defined as the error without directional bias, were computed^7^. Negative arithmetical difference represented an underestimation of the reproduced angle compared to the targeted angle shown by less knee extension and a positive value represented an overestimation of the angle with more knee extension. Moreover, the variable error (VE), representing the consistency of the constant error, was also calculated^22^.

### EMG recording and processing

For the surface EMG measurements, electrodes were placed on M. vastus medialis (VM), M. vastus lateralis (VL) and M. rectus femoris (RF) of both limbs, following the recommendations of SENIAM^23^. The skin was shaved, scraped with sandpaper and cleaned with alcohol for optimal detection of the muscle signals^20^. Bipolar electrodes (Type P-00-S, Blue Sensor®, Ambu, Denmark, inter-electrode distance: 20 mm) were used and the interelectrode impedance was kept below 2 kΩ (Impedance meter: D175, Digitimer®, Hertfordshire, UK). Data recordings were analogous to the description above. Neuromuscular activity during the JPS test was divided into four movement phases according to the angular recordings by the electrogoniometer: pre-activation, extension, isometric and flexion. The pre-activation was defined as the phase at the starting angle of 90°, 150 ms prior to initial knee extension movement towards the target angle. The extension phase was set from the start of extension until reaching the supposed target angle. A 500 ms timeframe in the middle of the three s holding at the supposed target angle was defined as the isometric phase. The flexion phase started from the first angular flexion movement towards the end of the extension/flexion movement to the initial starting angle of 90°.

Furthermore, EMG data were processed using Imago Process Master (Pfitec®, Endingen Germany). The raw signals were full wave rectified and band-pass filtered at 10–500 Hz (Butterworth, 2nd order). Amplitudes were calculated as root mean squares (RMS) for each muscle and movement phase and exported to a Microsoft® Excel spreadsheet (Windows 10, Microsoft Corporation, Redmond WA, USA). For the (submaximal) normalization of the neuromuscular activity, the mean gait cycle activity of each muscle during walking was used^20^.

### Statistical analysis

Statistical analyses were performed using R (R Core Team, version 4.1.0, 2021)^21^. Descriptive statistics were presented as median and interquartile range (IQR) of the constant, absolute as well as variable angular error and neuromuscular activity. Electrogoniometer and neuromuscular data were checked for plausibility. Individual values greater than two standard deviations from the grand mean were tracked back to original data. A verification of the data processing of these values was performed and adjusted if it was varying from the normal procedure or excluded from the dataset^20^.

For inferential statistics, the average values (angular error and neuromuscular activity) of the performed trials per block and session for each participant were calculated. The angular error and neuromuscular activity data were not normally distributed. Differences in angular error and neuromuscular activity (per muscle and movement phase) between leg dominance as well as sex, per block and session were assessed using Friedman-test for repeated measures (*α* = 0.05). This was performed to detect potential differences between and within the sessions. Additional effect sizes were calculated and classified as small (0.1–0.3), medium (0.3–0.5) or large (0.5–1.0)^24^. Moreover, to detect potential time-effects within the blocks of angular reproduction a spearman correlation between the count of repetition and the assessed errors was performed. The correlation was classified as weak (0.1–0.3), moderate (0.4–0.6), strong (0.7–0.9), perfect (1)^25^.

Reliability of the absolute angular error and neuromuscular activity for each muscle and phase was investigated within and between sessions. Relative reliability was calculated using intra class correlation coefficient (ICC; A,1) with 95% confidence intervals (CI)^26^ (R package irr (v0.84.1; Gamer, 2019)^27^). ICC values were classified as poor (< 0.5), moderate (0.5–0.75), good (0.75–0.9) and excellent (> 0.9)^28^. For absolute reliability a Bland Altman analysis (bias and limits of agreement (LoA)^29^ (R package blandr (v0.5.1; Datta, 2017)^30^) with the assessment of systematic and random error, and the calculation of the minimal detectable change (MDC: $\sqrt 2 *SEM$ [standard error of measurement; $SD*\surd 1 - ICC$]) were performed for intra- and inter-session reliability^31^.

### Ethics approval and consent to participate

The study complied with the criteria of the declaration of Helsinki and was ethically approved by the local legal authority Kantonale Ethikkommision für die Forschung (KEK No. 02200). All participants gave their informed consent prior to participation.

## Results

Twenty participants took part in this study (10 females and 10 males; age: 29 ± 8 years; height: 165 ± 39 cm; body mass 69 ± 13 kg). Mean weekly physical activity was approximately 260 min (4 h) with a standard deviation of 185 min (3 h). Median TAS was 4 with a range from 3 to 6. Participants performed a mean of 46 (range: 19–92) angular reproductions per leg and session.

### Angular error

**Figure 1:**
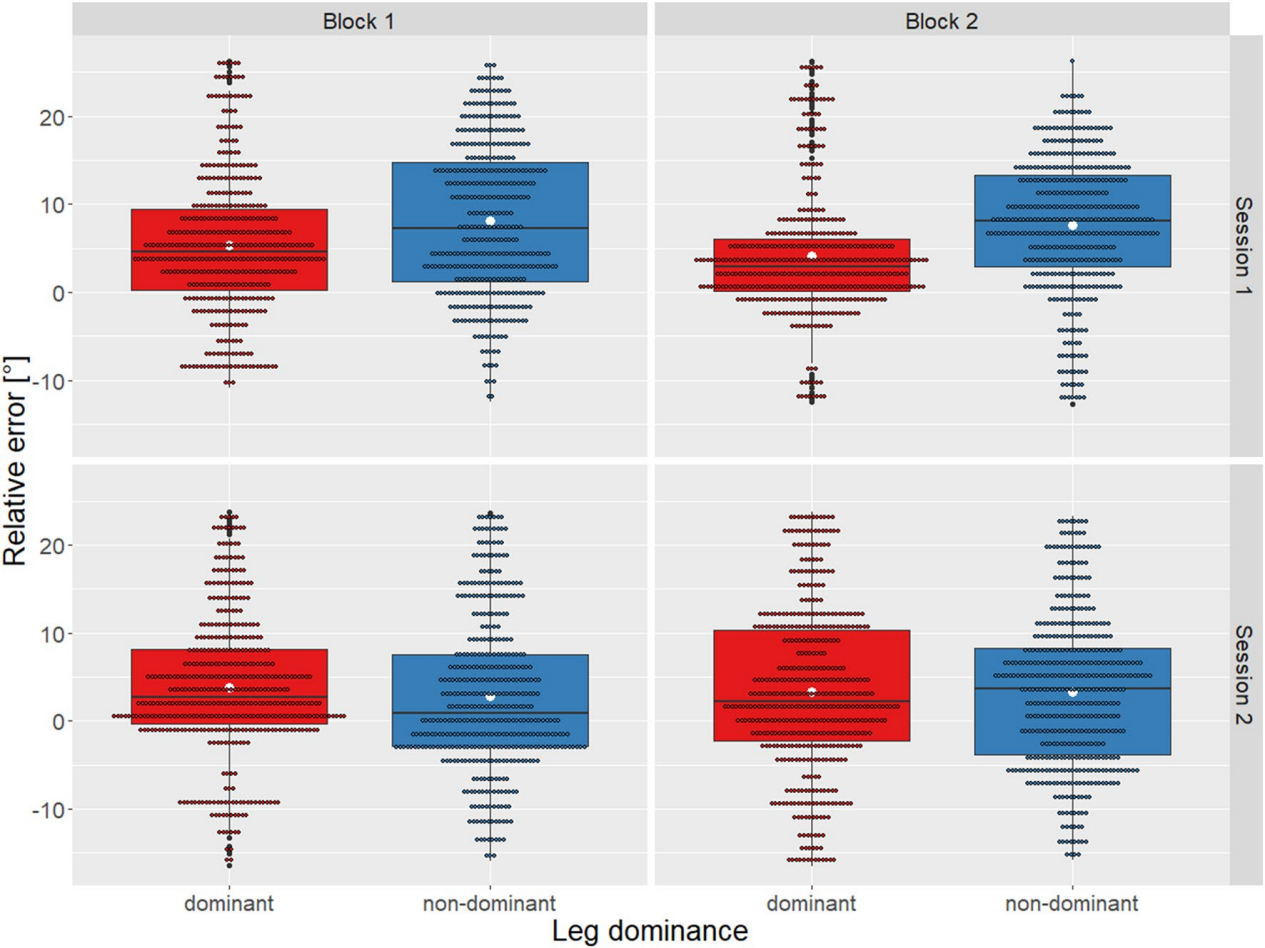
Constant error per leg dominance, session and block. Displayed as boxplots showing additional mean (white dot), outliers (bold black dot) and individual trials (small dot).

**Figure 2:**
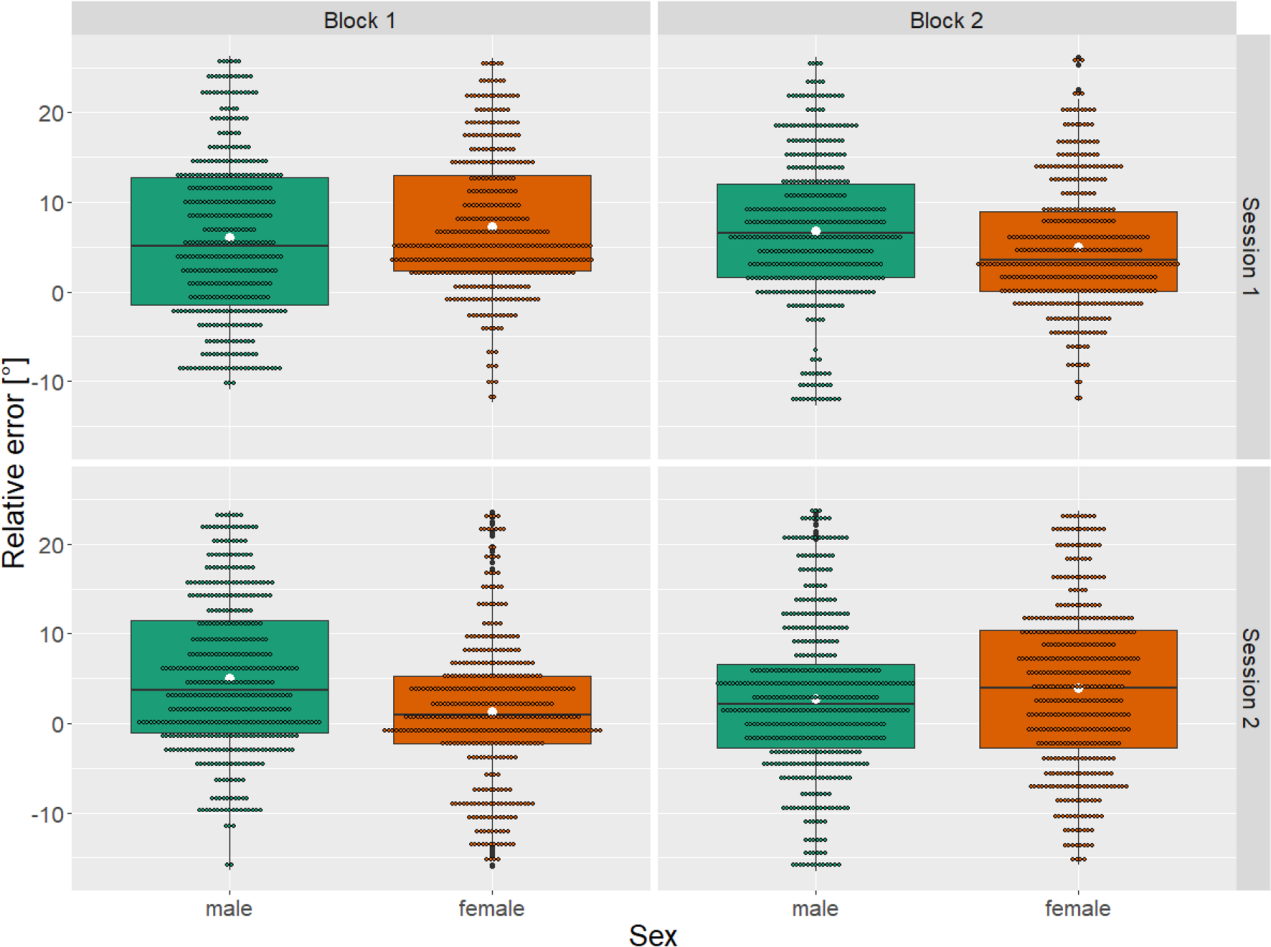
Constant error per sex, session and block. Displayed as boxplots showing additional mean (white dot), outliers (bold back dot) and individual trials (small dot).

The median (IQR) constant, absolute and variable angular error of all participants and sessions were 5.2° (6.9°), 6° (5.8°) and 0.99° (0.2°), respectively. For session one the constant, absolute and variable errors were 5.9° (7.8°), 6.8° (6.4°), 1° (0.1°), respectively. And for session two 3.4° (7.2°), 6.1° (4°), 0.9° (0.1°), accordingly. There was a weak correlation between the repetition count within the blocks and the errors (constant error: *r*_*s*_ = 0.01, *p* = 0.3; absolute error: *r*_*s*_ = 0.06, *p* = 0.0002; variable error: *r*_*s*_ = 0.004, *p* = 0.7). The constant errors according to leg dominance (Fig. 1) or sex (Fig. 2), per block and session are illustrated.

No significant difference between leg dominance or sex per blocks were found (*χ*^*2*^(7) = 8.508, *p* = 0.289, *r* =  − 0.07 and *χ*^*2*^(7) = 5.633, *p* = 0.58, *r* =  − 0.07, respectively). Median and IQR of the constant, absolute and variable error per leg dominance and session as well as sex and session are presented in Tables 1 and 2, respectively. Furthermore, in the supplemental information, constant, absolute and variable error in mean and standard deviation overall and per leg dominance, sex, session and block can be found (see Table S1).

**Table 1:** Constant, absolute and variable angular error displayed per leg dominance and session.

	Dominant leg		Non-dominant leg	
	*Session 1 M(IQR)*	*Session 2 M(IQR)*	*Session 1 M(IQR)*	*Session 2 M(IQR)*
Constant error [°]	3.7 (7.6)	2.5 (10)	7.9 (12)	2.3 (12)
Absolute error [°]	4.6 (6.4)	5.4 (8.9)	8.6 (10.3)	5.7 (7.5)
Variable error [°]	1 (0.2)	0.9 (0.1)	1.1 (0.2)	0.8 (0.2)

*M* median; *IQR* interquartile range.

**Table 2:** Constant, absolute and variable angular error displayed per sex and session.

	Female		Male	
	*Session 1 M(IQR)*	*Session 2 M(IQR)*	*Session 1 M(IQR)*	*Session 2 M(IQR)*
Constant error [°]	4.6 (9.9)	2 (10.8)	6.2 (11.9)	2.8 (11.3)
Absolute error [°]	4.9 (8.9)	6 (8)	7.6 (9.2)	5.3 (8.9)
Variable error [°]	1.1 (0.3)	0.9 (0.2)	1 (0.3)	0.9 (0.2)

*M* median; *IQR* interquartile range.

Inter-session reliability was poor (ICC = 0.424; 95% CI: 0.025–0.717) with a bias of 2.4° (± 2.4° LoA) and MDC of 6.8°. Moderate intra-session reliability (ICC = 0.723; 95% CI: 0.422–0.88) with a bias of 1.4° (± 1.65° LoA) and MDC of 4.7° were observed.

### Neuromuscular activity

**Figure 3:**
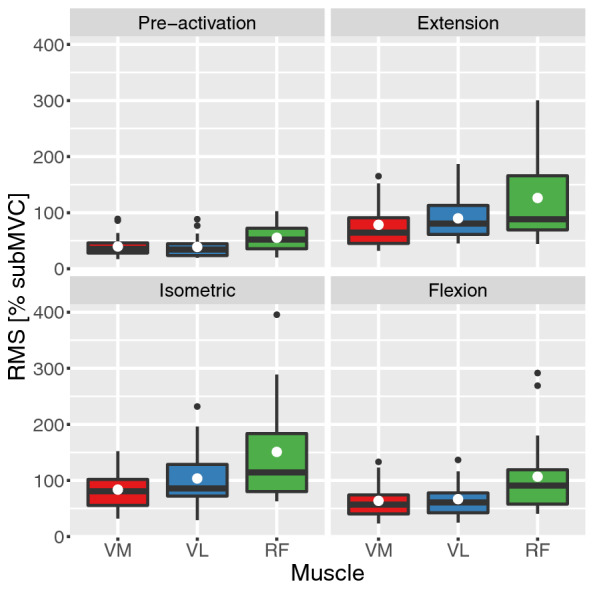
Neuromuscular activity per muscle and movement phase. Displayed as a boxplot with additional mean (white dot) and outliers (bold black dot). *RMS* root means square; *subMVC* submaximal voluntary contraction; *VM* vastus medialis; *VL* vastus lateralis; *RF* recuts femoris.

Neuromuscular activity for all assessed muscles and movement phases, summarized over both sessions and all blocks is illustrated in Fig. 3.

No significant differences between the dominant and non-dominant leg were found in any muscle and movement phase per blocks (0.024 < *p* < 0.779; 0.005 < *r* < 0.3; significant difference in the VM during flexion was not verified in the post-hoc analysis (*p* = 0.45)). Moreover, sex revealed no significant difference in any muscle and movement phase per blocks (0.059 < *p* < 0.824; 0.3 < r < 0.6). The median and IQR of the neuromuscular activity during the JPS test divided by the factors leg dominance and sex can be found in Tables 3 and 4, respectively. Additional information on mean and standard deviation of the overall neuromuscular activity and per leg dominance, sex, block and session can be found in the supplemental information (see Tables S2–4).

**Table 3:** Neuromuscular activity (% of the submaximal voluntary contraction) per leg dominance and session.

Phase	Muscle [%subMVC]	Dominant leg		Non-dominant leg	
		*Session 1 M(IQR)*	*Session 2 M(IQR)*	*Session 1 M(IQR)*	*Session 2 M(IQR)*
Pre-activation	VM	30.9 (18.6)	35.6 (18.1)	28.2 (19.9)	35.1 (22.1)
	VL	26.6 (16.7)	36.6 (30)	28.1 (19.8)	33.7 (23.1)
	RF	41.5 (41.5)	48.9 (40)	45.2 (40.4)	58.7 (52.6)
Extension	VM	66.9 (49.5)	51.9 (58.1)	71.6 (59.2)	61.7 (60.3)
	VL	79.5 (51.5)	60.6 (86.4)	81.5 (51.3)	76.5 (80.4)
	RF	76.8 (56.5)	80.8 (89.9)	74.7 (125.6)	79.6 (103)
Isometric	VM	80.6 (44)	62.7 (59.3)	75.5 (70.9)	75.3 (70.2)
	VL	104.2 (63.3)	82.7 (87.8)	78.8 (67.8)	85.9 (84)
	RF	79.7 (56.5)	97.4 (122.4)	106.5 (133.3)	91.8 (97)
Flexion	VM	53.8 (41)	45.8 (38.6)	60.6 (58.4)	46.5 (25.5)
	VL	63.4 (43.5)	46.9 (37.1)	52.4 (45.1)	49.1 (48.6)
	RF	76 (63.8)	74 (71.8)	84.3 (65.6)	79 (60.1)

*subMVC* submaximal voluntary contraction; *M* median; *IQR* interquartile range.

**Table 4:** Neuromuscular activity (% of the submaximal voluntary contraction) per sex and session.

Phase	Muscle [% subMVC]	Female		Male	
		*Session 1 M(IQR)*	*Session 2 M(IQR)*	*Session 1 M(IQR)*	*Session 2 M(IQR)*
Pre-activation	VM	30.3 (14.3)	35.3 (11.6)	28.8 (32.1)	35.9 (57.1)
	VL	29.1 (18.2)	36.4 (14.7)	26.2 (19.2)	29.1 (18.2)
	RF	36.5 (24.7)	40.3 (30.8)	55 (48.7)	63.9 (52.6)
Extension	VM	65.1 (37.7)	53.8 (25.3)	85 (79.9)	62.8 (80.1)
	VL	74.1 (39)	65.8 (51.8)	91 (54.7)	75.1 (106.6)
	RF	65.2 (35.5)	63.6 (32.7)	116.7 (140.6)	114.9 (111.1)
Isometric	VM	68.8 (41)	65.2 (34.2)	88.3 (83.1)	76.4 (89.4)
	VL	74.5 (56.9)	74.5 (58.5)	112.1 (67.1)	94.6 (135)
	RF	75 (39.5)	82.1 (45.8)	143.9 (160.1)	122.9 (166.9)
Flexion	VM	47.9 (25.1)	45.2 (14.5)	81.5 (72.5)	48 (65.7)
	VL	50.2 (34.1)	45.1 (19.5)	73.5 (45.8)	54.5 (71.7)
	RF	55 (47.3)	52.4 (39.4)	104.7 (81.3)	94.9 (75.1)

*subMVC* submaximal voluntary contraction; *M* median; *IQR* interquartile range.

Inter-session reliability measures differed between movement phase and muscle. ICC values ranged from 0.432 to 0.809, the bias between − 2.5 and 13.6% subMVC, and the MDC from 13.4 to 63.9% subMVC. Intra-session ICCs demonstrated moderate to excellent reliability ranging from 0.705 to 0.987, bias of − 7.7 to 2.4% subMVC and MDC ranging between 2.7 and 46.5% subMVC (Table 5).

**Table 5:** Reliability measures of the neuromuscular activity during the joint position sense test per movement phase and muscle.

Phase	Muscle	Inter-session			Intra-session		
		*ICC (95% CI)*	*Bias* ± *LoA*	*MDC*	*ICC (95% CI)*	*Bias* ± *LoA*	*MDC*
Pre-activation	VM	0.762 (0.49–0.898)	− 2.5 ± 29.2	15	0.986 (0.965–0.994)	0.9 ± 6.3	3.2
	VL	0.793 (0.55–0.913)	− 4.7 ± 24.7	13.4	0.987 (0.967–0.995)	− 0.4 ± 6.3	2.7
	RF	0.793 (0.554–0.912)	− 3.7 ± 32	26.3	0.959 (0.9–0.984)	− 0.2 ± 13	7.1
Extension	VM	0.472 (0.067–0.749)	13.6 ± 91.9	50	0.939 (0.855–0.975)	2.4 ± 33.5	17.6
	VL	0.432 (− 0.014–0.735)	8.8 ± 91.3	57.1	0.705 (0.375–0.875)	− 2.5 ± 63.5	38.1
	RF	0.845 (0.647–0.937)	13.2 ± 85.2	46.6	0.895 (0.74–0.959)	− 0.2 ± 72.2	39.8
Isometric	VM	0.688 (0.371–0.863)	6.3 ± 55.2	32.8	0.894 (0.755–0.956)	− 3.6 ± 33.3	19.7
	VL	0.688 (0.364–0.864)	6.2 ± 90.2	44.3	0.961 (0.905–0.984)	− 1.6 ± 34.8	15.2
	RF	0.819 (0.599–0.942)	− 7.9 ± 119.1	63.9	0.898 (0.764–0.958)	− 7.3 ± 89.4	46.5
Flexion	VM	0.655 (0.309–0.848)	12 ± 51.8	26.8	0.928 (0.829–0.971)	− 1 ± 29.6	15.1
	VL	0.688 (0.364–0.864)	6.2 ± 90.2	25.6	0.962 (0.905–0.984)	− 1.7 ± 34.8	15.2
	RF	0.809 (0.586–0.919)	6.2 ± 90.2	36.4	0.912 (0.791–0.965)	− 7.7 ± 65.9	30.4

*ICC* intra class correlation coefficient; *CI* confidence interval; *LoA* limits of agreement; *MDC* minimal detectable change.

## Discussion

The present study investigated the influence of leg dominance and sex on knee JPS measurements. Additionally, a reliability analysis was conducted regarding the angular reproduction performance as well as neuromuscular activity during an active knee JPS test in healthy participants. No significant differences between the dominant and non-dominant leg, nor between males and females were found. Reliability was poor to moderate with small systematic errors in angular reproduction performance and moderate to excellent in neuromuscular activity.

No significant differences in angular reproduction performance per leg dominance were found. This finding is in line with previous results, regardless of the methodological approach i.e., using passive or active repositioning^10,11^. Nonetheless, literature comparing the angular reproduction performance in trained and untrained participants reported differences in the dominant and non-dominant limbs^10,32^. Training may positively influence morphological adaptations of muscle spindles and induce adaptations in the central nervous system, e.g., increasing the strength of synaptic connections^10^. In the current study, participants were physically active as indicated by the Tegner activity scale and physical activity time per week. It is discussed in the literature, whether there is a positive causality between physical activity and JPS accuracy^33^. In the present study, no correlation analysis between physical activity or training load and JPS accuracy was performed. This might be an interesting avenue for future research, as deviations in physical activity and active time per week were high in the participants. Additionally, in the present study, no significant differences in neuromuscular activity per leg dominance were found. To the authors’ knowledge, no study has previously investigated neuromuscular activity during a JPS test, making comparisons difficult. Furthermore, determination of leg dominance lacks consensus^34^. Therefore, comparisons with other studies reporting limb dominance differences need to be conducted with caution.

Differences between males and females in JPS testing were not found in this study, confirming the findings of previous research^9,10^. Another study on sex differences in adolescents demonstrated significantly higher angular errors in females^35^. Nevertheless, it must be noted that age is regarded as an influencing factor, thus, a direct comparison to this study is not warranted^9,32^. Conversely, the age range of the present participants was rather narrow. A correlation between age and JPS accuracy was not performed and might be subject of future research. Furthermore, contrasts in neuromuscular activity of the quadriceps muscles between the sexes were not observed but paired with medium to high effect sizes. No literature was available examining sex differences in neuromuscular activity during a JPS test. The sample size in the current study was rather small, differences might become significant with more participants.

The detected homogeneity in angular reproduction performance and neuromuscular activity between limb dominance and sex can justify overall analysis of reliability measures without further subdivision. Relative inter-session reliability for the angular error was poor. A single study has previously reported similar or slightly better results for reliability measures. They identified ICC scores of 0.24 to 0.69 in an active seated JPS test in three different target angles^36^. Another study reported moderate inter-rater reliability in a seated JPS test using an electrogoniometer^37^. Relative reliability of the electrogoniometer did not reach the excellent results demonstrated in a study comparing different knee goniometry devices^38^. Limited inter-session ICC scores and better reproduction performance in the present study might be due to a learning effect. Although a separation of two weeks between the measurement sessions was chosen, the second session may show better results due to a familiarization with the task. Absolute inter-session reliability with a bias of 2.4° (± 2.4° LoA) and MDC of 6.8° was in the range of systematic errors found in another study reporting a standard error of measurement of 1.4–2.9°^37^. Moreover, the reported MDC exceeded the minimal difference for clinical relevance of 5° assumed by the literature^13^. This must be kept in mind when using this assessment in a pathological population. Nevertheless, the reported MDC seems to be in an acceptable range when compared to other angular measurement devices^38^.

Differences in reliability measures in this study compared to others may have been elicited by the study protocol. The participants performed two blocks of 3-min of angular reproduction at a self-selected pace. Hereby, on average 46 repetitions were performed while other studies reported three to ten repetitions in their examination^36,37^. Thus, greater variability in reliability measures might be attributed to the time between familiarization trials and ongoing reproduction, or because of possible fatigue. However, the weak correlation of the repetition count and the errors demonstrated no time-effect within the blocks. To reduce a potential increase of errors due to fatigue, the JPS test was divided into two blocks per session. The moderate intra-session ICC scores and absolute reliability values, not exceeding the values of the inter-session comparison, showed that the separation might prevent from major fatigue and does not enhance learning effects over the whole session. Therefore, the blocks of reproduction can be summarized during the data analysis and collectively provide sufficient repetitions to accurately determine the angular reproduction performance^39^.

Relative inter- and intra-session reliability of the neuromuscular activity were rated moderate to excellent except for the inter-session ICC score during extension, which was poor. Comparisons of the absolute reliability, especially the MDC, did not show considerable variation between the movement phases. A possible explanation for the limited relative reliability during extension might be a change in the movement velocity with ongoing repetitions. To the authors’ knowledge, no studies investigating the reliability of neuromuscular activity during a JPS test have been published.

In a clinical setting, the use of an (electro)goniometer represents an inexpensive and user-friendly method that can be assessed by a single operator. The described findings of the neuromuscular activity paired with the results of the angular reproduction performance, especially the MDC, can further be used for comparisons to a pathological population, to verify clinically relevant changes e.g., throughout rehabilitation process.

This study has certain limitations. The targeted angle was in the range of typical daily activities and was previously used in the literature^40,41^. However, only one angle and movement direction were represented. Further variations of angles and movement directions might give more precise insights. Additionally, the performed JPS task does not reflect comprehensive sensorimotor performance during a functional task with multi-joint movements. Differences between limb dominance and sex, in both sensorimotor performance as well as neuromuscular activity, could potentially have been elicited in a more challenging task. The clinical relevance of a JPS task has been questioned in the literature^42,43^. A weight-bearing JPS test or kinaesthetic movement reproduction task representing sensorimotor performance in a more functional setting has been proposed^44,45^. Nevertheless, no gold standard could be identified^46^. Evaluations containing functional weight-bearing tasks might not be feasible during the acute phase of injury. In this case, a non-weight-bearing open kinetic chain task to detect side asymmetries could be an option to monitor progress. Yet, a newly introduced obstacle clearance test might fill the gap between the limitations of a JPS test and a functional task, also in acute patients^33^. Further research on the development of a sensorimotor performance assessment technique is needed^2^.

Moreover, the participants were allowed to perform the JPS test at a self-selected pace resulting in a wide range of repetitions. As mentioned above, the movement velocity might influence the recorded angular reproduction performance and neuromuscular activity.

During data analysis, it was identified that participants were not always holding the supposed target angle isometrically, resulting in a certain variation of the angle over the holding period. Nevertheless, the turnaround points of knee extension maximum and starting of flexion during the reproduction task were distinct. For reproducibility reasons always the midpoint between the two turnarounds represented the reproduced angles.

Another limitation might be the long duration of angular reproduction with two blocks of three minutes. Participants might have lost focus and motivation. Nonetheless, the small variable errors paired with a weak correlation between the repetition count within the blocks and the assessed errors reflect no great variation during the execution of the task. To minimize this possible issue a break between the blocks was included in the protocol. Moreover, the high number of repetitions assured an accurate evaluation of the angular reproduction performance, speaking in favour of the chosen reproduction time^39^.

## Conclusions

The active knee joint position sense test demonstrated variable reliability measures from poor to excellent paired with a consistency of measurement in an acceptable range. Findings assume no influences of leg dominance and sex on the angular reproduction performance and neuromuscular activity. Despite these results, comparisons between different populations e.g., healthy participants and individuals with a pathology should be conducted with caution. Practitioners can use the JPS test as a tool for assessing sensorimotor function over the course of the rehabilitation process, but it should be interpreted considering the mentioned limitations.

## Supplementary Information

Supplementary Information.

## Data Availability

All data generated or analysed during this study are included in this published article and its supplementary information files.
