# Supplementary material for: Reliability of the active knee joint position sense test and influence of limb dominance and sex
Source: Sci Rep. 2023 Jan 4;13:152. doi: 10.1038/s41598-022-26932-2 (PMC9813362; doi:10.1038/s41598-022-26932-2)
Supplement: Supplementary file 1 — Supplementary Information. [file 41598_2022_26932_MOESM1_ESM.pdf]

Supplementary Information

Influence of leg dominance, sex and reliability on the active knee joint position sense test in healthy adults

Aglaja Busch [1,2*], Christian Bangerter [2], Frank Mayer [1], Heiner Baur [2]

[1] University Outpatient Clinic, Sports Medicine & Sports Orthopeadics, University of Potsdam, Am Neuen Palais 10, 14469 Potsdam, Germany

[2] Division of Physiotherapy, Department of Health Professions, Bern University of Applied Sciences, Murtenstrasse 10, 3008 Bern, Switzerland

Table S1 Constant, absolute and variable error overall and per leg dominancy and sex for each session and trial.

		Session 1		Session 2	
		Block 1 Mean±SD	*Block 2 Mean±SD*	*Block 1 Mean±SD*	*Block 2 Mean±SD*
**Overall**	Constant error [°]	6.6±8.4	5.8±7.6	3.3±8.3	3.3±9
	Absolute error [°]	8.4±6.6	7.4±6	6.7±5.9	7.6±5.9
	Variable error [°]	1.1±0.2	1±0.2	0.9±0.1	0.9±0.1
**Dominant leg**	Constant error [°]	5.2±8	4±7.2	3.8±7.9	3.3±9.4
	Absolute error [°]	7.5±6	5.6±6	6.5±5.9	7.7±6.3
	Variable error [°]	1±0.2	0.9±0.2	0.9±0.1	0.9±0.1
**Non-dominant leg**	Constant error [°]	8±8.6	7.5±7.6	2.8±8.8	3.2±8.6
	Absolute error [°]	9.4±7.1	9.2±5.5	7±6	7.4±5.4
	Variable error [°]	1.1±0.2	1.1±0.3	0.9±0.2	0.9±0.2
**Female**	Constant error [°]	7.2±7.6	4.9±6.9	1.3±7.7	3.9±9.2
	Absolute error [°]	8.1±6.7	6.3±5.7	5.8±5.3	8.2±5.8
	Variable error [°]	1.1±0.2	1±0.2	0.9±0.1	0.9±0.2
**Male**	Constant error [°]	6±9.1	6.7±8.1	5±8.3	2.6±8.8
	Absolute error [°]	8.7±6.5	8.5±6.2	7.5±6.3	7±5.9
	Variable error [°]	1±0.2	1±0.2	0.9±0.1	0.9±0.1

SD = standard deviation

Table S2 Overall neuromuscular activity per muscle, session, block and movement phase.

**Muscle**	**Session**	**Block**	**Pre-activation [% subMVC]**		**Extension [% subMVC]**		**Isometric [% subMVC]**		**Flexion [% subMVC]**	
			Mean	SD	Mean	SD	Mean	SD	Mean	SD
**RF**	1	1	51.9	22.9	127.6	78.5	143.9	97.4	107.1	70.3
**RF**	1	2	50.8	22.0	121.2	81.3	146.5	107.0	107.7	86.3
**RF**	2	1	57.1	27.7	117.2	79.2	155.0	103.3	101.0	67.8
**RF**	2	2	54.7	25.5	125.5	91.6	147.3	104.1	101.3	80.9
**VL**	1	1	34.0	16.4	89.6	44.3	104.3	60.5	67.8	26.4
**VL**	1	2	36.8	18.8	96.2	41.6	109.0	64.8	73.5	40.2
**VL**	2	1	41.1	21.4	86.2	48.1	101.1	54.8	62.8	30.6
**VL**	2	2	40.8	21.3	82.7	47.7	102.0	54.9	62.5	32.7
**VM**	1	1	38.7	19.5	83.5	49.6	84.5	33.6	69.6	36.5
**VM**	1	2	37.7	19.3	81.1	47.2	88.2	40.0	70.7	41.8
**VM**	2	1	40.9	22.8	69.9	42.1	78.1	37.6	57.5	30.1
**VM**	2	2	41.8	24.4	74.7	45.8	83.6	39.5	59.3	30.1

RF = rectus femoris; VL = vastus lateralis; VM = vastus medialis; subMVC = submaximal voluntary contraction; SD = standard deviation

Table S3 Neuromuscular activity per leg dominance, muscle, session, block and movement phase.

Leg dominance	Muscle	Day	Trial	Pre-activation [% subMVC]		Extension [% subMVC]		Isometric [% subMVC]		Flexion [% subMVC]	
				Mean	SD	Mean	SD	Mean	SD	Mean	SD
Dominant	RF	1	1	51.0	29.0	116.6	91.5	127.6	94.9	89.6	58.7
	RF	1	2	48.4	23.6	115.5	101.7	124.7	122.9	93.5	75.5
	RF	2	1	51.7	23.9	108.7	74.2	138.5	105.7	85.3	43.8
	RF	2	2	53.0	22.8	120.1	88.9	146.6	117.6	97.6	74.7
	VL	1	1	30.3	11.7	75.5	30.4	99.3	43.3	66.3	29.7
	VL	1	2	33.6	16.2	87.5	63.1	110.0	68.8	74.2	44.5
	VL	2	1	40.3	22.7	78.6	50.9	91.5	53.6	59.0	31.3
	VL	2	2	42.1	23.4	91.3	67.6	104.5	72.9	63.8	40.7
	VM	1	1	36.3	18.6	72.2	36.4	80.6	31.6	64.3	32.7
	VM	1	2	36.9	17.0	64.8	33.7	76.0	34.3	57.9	29.9
	VM	2	1	42.6	25.2	64.0	41.2	69.2	37.6	55.0	30.7
	VM	2	2	43.5	27.2	69.2	43.8	76.3	39.1	56.4	30.3
Non-dominant	RF	1	1	51.9	22.2	140.1	95.0	165.8	102.4	124.0	73.8
	RF	1	2	50.8	24.6	114.7	86.1	157.2	105.5	111.9	91.7
	RF	2	1	60.0	38.7	125.3	103.9	152.6	120.9	103.1	78.1
	RF	2	2	55.6	28.7	116.6	93.6	136.1	99.3	91.5	70.6
	VL	1	1	30.7	16.8	102.1	67.6	98.5	68.9	64.9	29.1
	VL	1	2	32.0	16.3	102.4	69.6	95.9	64.6	61.3	29.3
	VL	2	1	35.6	20.3	85.0	48.7	98.4	59.7	62.2	37.5
	VL	2	2	38.2	22.6	85.3	44.3	104.7	59.5	63.4	34.6
	VM	1	1	33.6	19.8	84.2	57.1	87.8	43.6	65.6	30.6
	VM	1	2	32.8	21.3	75.5	42.9	93.1	53.7	68.7	41.5
	VM	2	1	32.7	18.6	65.6	38.5	78.7	40.8	50.7	24.1
	VM	2	2	39.0	26.0	81.0	54.0	97.4	55.7	60.0	34.4

RF = rectus femoris; VL = vastus lateralis; VM = vastus medialis; subMVC = submaximal voluntary contraction; SD = standard deviation

Table S4 Neuromuscular activity per sex, muscle, session, block and movement phase.

Sex	Muscle	Session	Block	Pre-activation [% subMVC]		Extension [% subMVC]		Isometric [% subMVC]		Flexion [% subMVC]	
				Mean	SD	Mean	SD	Mean	SD	Mean	SD
**Male**	RF	1	1	58.0	26.7	153.1	79.2	175.7	105.0	122.6	68.0
	RF	1	2	57.3	27.9	155.5	75.1	185.7	107.8	127.4	95.2
	RF	2	1	65.5	32.5	146.5	80.4	179.9	100.4	115.5	64.0
	RF	2	2	64.3	30.0	158.9	90.3	170.3	91.1	113.4	73.6
	VL	1	1	36.2	21.3	104.6	54.6	117.8	78.9	72.5	24.9
	VL	1	2	41.2	24.3	120.0	41.1	131.8	79.2	88.3	47.4
	VL	2	1	46.8	27.1	98.9	58.7	117.9	66.4	69.4	36.8
	VL	2	2	47.3	27.7	88.3	58.9	114.6	68.0	70.1	40.1
	VM	1	1	44.4	25.1	101.3	63.4	92.0	40.8	83.1	45.5
	VM	1	2	43.4	25.5	101.7	57.7	101.8	47.4	89.2	51.2
	VM	2	1	47.6	30.5	83.1	51.8	87.5	46.0	68.5	39.3
	VM	2	2	48.6	33.1	86.0	58.0	89.8	50.3	70.3	39.7
**Female**	RF	1	1	46.4	18.6	104.6	74.1	115.3	85.3	93.2	72.9
	RF	1	2	45.5	15.5	93.8	79.0	115.2	100.7	91.8	79.8
	RF	2	1	48.7	20.1	88.0	69.7	130.1	105.3	86.4	71.6
	RF	2	2	45.2	16.4	92.0	84.1	124.3	115.8	89.2	89.8
	VL	1	1	32.0	11.0	76.2	29.3	92.2	38.0	63.5	28.2
	VL	1	2	32.5	10.7	72.4	26.4	86.3	38.1	58.7	26.1
	VL	2	1	35.3	12.3	73.5	32.9	84.2	36.2	56.2	22.9
	VL	2	2	34.3	9.5	77.2	35.4	89.5	37.2	55.0	22.8
	VM	1	1	32.9	10.0	65.8	21.8	77.1	24.5	56.1	18.3
	VM	1	2	32.0	7.9	60.5	20.6	74.6	26.7	52.1	17.4
	VM	2	1	34.2	8.3	56.7	25.9	68.8	26.0	46.5	10.3
	VM	2	2	35.0	7.6	63.5	27.8	77.4	25.9	48.3	8.1

RF = rectus femoris; VL = vastus lateralis; VM = vastus medialis; subMVC = submaximal voluntary contraction; SD = standard deviation
